# LOTUS suppresses amyloid β-induced dendritic spine elimination through the blockade of amyloid β binding to PirB

**DOI:** 10.1186/s10020-022-00581-7

**Published:** 2022-12-12

**Authors:** Yuki Kawaguchi, Junpei Matsubayashi, Yutaka Kawakami, Ryohei Nishida, Yuji Kurihara, Kohtaro Takei

**Affiliations:** 1 Molecular Medical Bioscience Laboratory, Department of Medical Life Science, Yokohama City University Graduate School of Medical Life Science, 1-7-29 Suehiro-Cho, Tsurumi Ward, Yokohama, 230-0045 Japan; 2 Department of Anesthesiology, National Center of Neurology and Psychiatry, Kodaira, Japan; 3 Department of Molecular Biology, Graduate School of Pharmaceutical Sciences, Nagoya City University, Nagoya, Japan

**Keywords:** Amyloid beta protein, Alzheimer’s disease, Paired immunoglobulin-like receptor B, Lateral olfactory tract usher substance, Cofilin, Post-synaptic density-95, Spine, Leukocyte immunoglobulin-like receptor subfamily B member 2

## Abstract

**Background:**

Alzheimer’s disease (AD) is the most common neurodegenerative disease worldwide but has no effective treatment. Amyloid beta (Aβ) protein, a primary risk factor for AD, accumulates and aggregates in the brain of patients with AD. Paired immunoglobulin-like receptor B (PirB) has been identified as a receptor of Aβ and Aβ–PirB molecular interactions that cause synapse elimination and synaptic dysfunction. PirB deletion has been shown to suppress Aβ-induced synaptic dysfunction and behavioral deficits in AD model mice, implying that PirB mediates Aβ-induced AD pathology. Therefore, inhibiting the Aβ–PirB molecular interaction could be a successful approach for combating AD pathology. We previously showed that lateral olfactory tract usher substance (LOTUS) is an endogenous antagonist of type1 Nogo receptor and PirB and that LOTUS overexpression promotes neuronal regeneration following damage to the central nervous system, including spinal cord injury and ischemic stroke. Therefore, in this study, we investigated whether LOTUS inhibits Aβ–PirB interaction and Aβ-induced dendritic spine elimination.

**Methods:**

The inhibitory role of LOTUS against Aβ-PirB (or leukocyte immunoglobulin-like receptor subfamily B member 2: LilrB2) binding was assessed using a ligand-receptor binding assay in Cos7 cells overexpressing PirB and/or LOTUS. We assessed whether LOTUS inhibits Aβ-induced intracellular alterations and synaptotoxicity using immunoblots and spine imaging in a primary cultured hippocampal neuron.

**Results:**

We found that LOTUS inhibits the binding of Aβ to PirB overexpressed in Cos7 cells. In addition, we found that Aβ-induced dephosphorylation of cofilin and Aβ-induced decrease in post-synaptic density-95 expression were suppressed in cultured hippocampal neurons from LOTUS-overexpressing transgenic (LOTUS-tg) mice compared with that in wild-type mice. Moreover, primary cultured hippocampal neurons from LOTUS-tg mice improved the Aβ-induced decrease in dendritic spine density. Finally, we studied whether human LOTUS protein inhibits Aβ binding to LilrB2, a human homolog of PirB, and found that human LOTUS inhibited the binding of Aβ to LilrB2 in a similar manner.

**Conclusions:**

This study implied that LOTUS improved Aβ-induced synapse elimination by suppressing Aβ-PirB interaction in rodents and inhibited Aβ–LilrB2 interaction in humans. Our findings revealed that LOTUS may be a promising therapeutic agent in counteracting Aβ-induced AD pathologies.

## Introduction

Alzheimer’s disease (AD) is one of the most devastating neurodegenerative diseases that is characterized by senile plaque and neurofibrillary tangle. Amyloid beta (Aβ) protein was found to be a critical factor in neurodegeneration observed in the brain of patients with AD and the initial catalyst for other AD pathologies in several studies on postmortem human brain tissue (Glenner and Wong 1980; Hardy and Higgins 1992). Aβ is cleaved from amyloid precursor protein by β-secretase and γ-secretase. Aβ peptide is composed of 40–42 amino acids and tends to form oligomers owing to its high-aggregation propensity. Oligomeric Aβ peptide forms amyloid plaque so as to interact with several cell surface receptors, leading to synaptic dysfunction, neuronal loss, and neuroinflammation, which is a contributing factor to cognitive impairment caused by AD (Gómez-Isla et al. 1997; Mucke et al. 2000; Okello et al. 2009; Querfurth and LaFerla 2010).

More than a dozen receptors have been reported as Aβ receptors so far, including cellular prion protein (PrPC), Nogo receptor type 1 (NgR1), p75 neurotrophin receptor (p75NTR), and paired immunoglobulin-like receptor B (PirB) (Lauren et al. 2009; Kim et al. 2013; Zhao et al. 2017a, b; Yaar et al.^1997^; Smith et al. 2019). PrPC localizes at the postsynaptic membrane, and Aβ binding to PrPC activates Fyn kinase and the subsequent phosphorylation and aberrant cell surface localization of the NR2B, a subunit of *N*-methyl-d-aspartate receptors, leading to the calcium influx and cytotoxicity (Um et al. 2012). PrPC deletion mice or anti-PrPC therapy enhances Aβ-induced Fyn activation and neuronal cell death (Um et al. 2012). The Nogo receptor family, NgR1-3, is reported as a negative regulator of synapse assembly and plasticity (Mironova and Giger 2013). NgR1 has recently been reported as an Aβ receptor, and the suppression of NgR1 expression by short hairpin RNA interference has been found to attenuate Aβ-induced dendritic spine loss (Zhao et al. 2017a, b). p75NTR, one of the important neurotrophin receptors that play a crucial role in neuronal development primarily as a co-receptor of several membranous receptors in the central nervous system (CNS), was also identified to be another Aβ receptor (Yaar et al. 1997). Aβ-induced dendritic spine loss was suppressed in cultured neurons obtained from p75NTR knock-out mice compared with neurons obtained from wild-type (WT) mice. Moreover, TAT-Pep5, particularly inhibits p75NTR suppressed Aβ-induced dendritic spine loss in cultured neurons (Patnaik et al. 2020).

However, a previous study demonstrated that PirB functions as a receptor for Aβ and downregulates neuronal plasticity via cofilin dephosphorylation and PSD95 downregulation. These intracellular alterations result in severe cognitive impairment (Kim et al. 2013). In contrast, PirB-deficient mice demonstrate more improved dendritic spine formation and cognitive function in comparison with WT mice (Vidal et al. 2016; Djurisic et al. 2019; Albarran et al. 2021). PirB and its human homolog leukocyte immunoglobulin-like receptor subfamily B member 2 (LilrB2) were initially known as inhibitory receptors in immune cells, including B cells, mast cells, macrophages, and dendritic cells (Takai 2005). However, PirB was also found to be expressed in CNS neurons and identified as a receptor for myelin-associated inhibitors (MAIs), including Nogo-A, myelin-associated glycoprotein, and oligodendrocyte myelin glycoprotein (Syken et al. 2006; Atwal et al. 2008). These results imply that Aβ binding to its receptors is the primary culprit of Aβ-induced neuronal degeneration and that regulation of Aβ receptor function is anticipated to be efficient for neutralizing AD pathogenesis.

Among these receptors for Aβ, we paid attention to PirB-mediated neuronal dysfunction because PirB may mediate both Aβ-induced neuronal degeneration and MAI-induced axon growth inhibition. Moreover, it has been reported that PirB has considerably greater Aβ binding engagement than NgR1 (Smith et al. 2019). Lateral olfactory tract usher substance (LOTUS) is expressed on the cell surface in the CNS. It plays an important role in the formation of lateral olfactory tract by inhibiting NgR1 activity (Sato et al. 2011). LOTUS suppresses neurite outgrowth inhibition caused by the binding of all five types of ligands to NgR1 (Kurihara et al. 2014, 2017; Kawakami et al. 2018a, b). Thus, LOTUS induces neuronal regeneration via blockade of NgR1 function in spinal cord injury (Hirokawa et al. 2017; Ito et al. 2018, 2021), brain ischemia (Takase et al. 2017), optic nerve injury (Hirokawa et al. 2017; Kawakami et al. 2018a), and unilateral pyramidotomy (Ueno et al. 2020). Moreover, LOTUS influences synapse formation and cognitive function in vivo (Nishida et al. 2021). Recently, we found that LOTUS also binds to PirB and suppresses its function (Kurihara et al. 2020). These studies implied that LOTUS acts as an endogenous antagonist for not only NgR1 but also PirB, thereby increasing the possibility that LOTUS acts as an inhibitory regulator for axon growth inhibition and neuronal degeneration. Nevertheless, whether LOTUS also suppresses the Aβ-induced neurodegenerative effects mediated by PirB remains unelucidated. Interestingly, it has been reported that LOTUS is gradually decreased in the hippocampus with aging (Starkey et al. 2013). The decrease in LOTUS with aging may be expected to increase Aβ-PirB binding and action, leading to an increased risk of AD.

Herein, we demonstrated that LOTUS inhibited the binding of Aβ to PirB, Aβ-induced cofilin dephosphorylation and PSD95 downregulation, and Aβ-induced decrease in dendritic spine density. We also confirmed that human LOTUS interacts with the human homolog of PirB LilrB2 and inhibits Aβ–LilrB2 binding. The results suggest that LOTUS acts as an endogenous suppressor against Aβ–PirB interaction, thereby inhibiting Aβ-induced spine elimination.

## Methods

### Animals

C57BL/6J WT mice (RRID: IMSR_JAX: 000664) were bought from SLC. LOTUS-overexpressing transgenic (LOTUS-tg) mice were created using synapsin-1 promoter, which selectively expresses neuron-specific LOTUS, as previously explained (Hirokawa et al. 2017). These mice were raised in rectangular plastic cages with stainless-steel mesh covers (four mice in each cell at most) in a pathogen-free facility under 12 h/12 h light/dark conditions and free access to autoclaved water and food. During the experimental procedures, all efforts were made to reduce the number of animals used and minimize their suffering. The experimental procedures were approved by the institutional animal care and use ethical committee of Yokohama City University and were conducted in accordance with the approved guidelines. The procedures were approved by the institutional animal care and use ethics committee of Yokohama City University (approval number #T-A-20-002).

### Antibodies and reagents

Mouse monoclonal antibodies against rat LOTUS (custom-made, ITM, RRID: AB_2819118), goat polyclonal antibodies against mouse PIR-B (AF2754, R&D Systems, RRID: AB_2249965), rat monoclonal antibodies against mouse PIR-B (550348, BD Biosciences, RRID: AB_393627), mouse monoclonal antibodies against synthetic β-actin (A5316, Sigma-Aldrich RRID: AB_476743), rabbit monoclonal antibodies against cofilin (#5175, CST, RRID: AB_10622000), rabbit monoclonal antibodies against phospho-cofilin (Ser3) (77G2) (#3313, CST, RRID: AB_2080597), mouse monoclonal antibodies against PSD-95 (MA1-046, Thermo Fisher Scientific, RRID: AB_2092361), goat polyclonal antibody against human immunoglobulin (Ig)-like transcript 4 (ILT4)/CD85d (AF2078, R&D systems, RRID: AB_355136), biotin-SP-labeled goat antibodies against mouse IgG (115-065-003, Jackson ImmunoResearch, RRID: AB_2338557), Alexa Fluor 488-labeled donkey antibodies against mouse IgG (715-545-151, Jackson ImmunoResearch, RRID: AB_2341099), Alexa Fluor 596-labeled donkey antibodies against goat IgG (705-585-147, Jackson ImmunoResearch, RRID: AB_2340433), purified rat IgG (6-001-A, R&D Systems, RRID: AB_10144734), peroxidase-conjugated antibodies against mouse IgG (115-035-003, Jackson ImmunoResearch, RRID: AB_10015289), peroxidase-conjugated antibodies against rabbit IgG (111-035-003, Jackson ImmunoResearch, RRID: AB_2313567).

### Aβ preparation

Recombinant biotin-LC-Aβ (1–42) peptides (AS-20276, Anaspec Inc) were dissolved in 1% NH_4_OH and diluted in phosphate-buffered saline (PBS) to the concentration of 100 µM. The Aβ peptide was incubated at 37 °C for 48 h and stored at − 80 °C until use for Aβ to oligomerize. Successful Aβ oligomerization was confirmed by non-reduced sodium dodecyl sulfate-polyacrylamide gel electrophoresis (SDS-PAGE).

### Construction of plasmid vectors

Plasmids encoding full-length mouse LOTUS (NM_145123) and full-length mouse PirB (NM_001357394) were generated as previously explained (Sato et al. 2011; Kurihara et al. 2020). The plasmid encoding enhanced green fluorescent protein (EGFP) was generated by injecting the coding sequence of the EGFP construct (pEGFP-N3, 6080-1, BD Bioscience) into the 3′ region of the CAG promoter in another mammalian exhibition vector. The plasmids encoding full-length human LilrB2 (NM_001080978) and full-length human LOTUS (NM_001206528) construct were generated by subcloning the full-length cDNA of LilrB2 and LOTUS from the human brain cDNA library (9503, TAKARA) into the vector. The expression plasmids of the mutated human Fc-tagged Streptavidin binding protein (SBP) (Fc-SBP), original signal peptide region-, and the transmembrane region-deleted mouse LOTUS fusing Fc-SBP (LOTUS-Fc-SBP), as previously described (Kurihara et al. 2020). Correct alignment of the nucleotides inserted into each plasmid was validated by DNA sequencing.

### Ligand-receptor binding assay and immunocytochemistry

Cos-7 cells (RRID: CVCL_0024) cells were cultured in Dulbecco’s modified eagle’s medium (DMEM) (08458-16, Nacalai Tesque) including 10% fetal bovine serum (FBS) (04-001-1A, Biological Industries) and 0.5% penicillin–streptomycin mixed solution (Nacalai Tesque). All cells were operated using a sterile cell culture procedure and incubated at 37 °C with 5% CO_2_. Cells were seeded at 5.0 × 10^4^ cells/well in 4-well culture plates (176740, Thermo Fisher Scientific). The plasmids encoding mouse LOTUS, mouse PirB, human LOTUS, or human LilrB2 were transfected to the cells using FuGENE 6 (E2691, Promega) and cultured for 44 h. For the binding assay, transfected cells were treated with biotin-LC-Aβ (1–42) at each concentration for 1 h at 37 °C with 5% CO_2_. Following treatment, the cells were fixed with 4% PFA in PBS containing 2 mM MgCl_2_ for 1 h at room temperature (RT), rinsed with PBS containing 2 mM MgCl_2_, and incubated for 1 h at 67 °C to inactivate endogenous alkaline phosphatase (AP). Next, the cells were incubated with AP-conjugated avidin–biotin complex (AK-5000, Vector Laboratories, RRID: AB_2336792) in TBS-T for 1 h at RT. Aβ peptide bound to the cells was visualized with the enzymatic reaction product of nitro blue tetrazolium (NBT) (11383213001, Roche) and 5-bromo-4-chloro-3-indolyl phosphate (BCIP) (11383221001, Roche). The digital images were captured with a BZ-8100 microscope (Keyence) fitted with a 10× objective lens. For the purpose of quantification, its intensity was detected using pNPP (N2770-50SET, Sigma-Aldrich) by measuring its absorbance at 405 nm using xMark Microplate Absorbance Spectrophotometer (Bio-Rad) and Microplate Manager 6 software (Bio-Rad).

Immunocytochemistry was used to validate the cell surface expression of mouse PirB and human LilrB2 under non-permeabilizing conditions as previously described (Sato et al. 2011; Kurihara et al. 2012, 2014, 2020). Briefly, the transfected and cultured cells were incubated with antibodies against mouse PIR-B (1 µg/ml, AF2754, R&D Systems, RRID: AB_2249965), human ILT4/CD85d (1 µg/ml, AF2078, R&D systems, RRID: AB_355136) and/or LOTUS (1 µg/ml, H24G11-mAb, Sato et al. 2011, RRID: AB_2819119) in the culture medium for 1 h at 37 °C with 5% CO_2_ and subsequently fixed with 4% PFA in PBS for 1 h at RT. After washing with TBS-T, the fixed cells were incubated with Alexa Fluor 488-labeled donkey antibodies against mouse IgG (0.75 µg/ml, 715-545-151, Jackson ImmunoResearch, RRID: AB_2341099) and Alexa Fluor 596-labeled donkey antibodies against goat IgG (0.75 µg/ml, 705-585-147, Jackson ImmunoResearch, RRID: AB_2340433) in TBS-T for 1 h at RT. Digital images were captured with a BZ-8100 microscope (Keyence) equipped with a 10× objective lens. For quantitative detection of cell surface expression of PirB or LilrB2, the transfected and cultured cells were incubated with antibodies against PIR-B (1 µg/ml, AF2754, R&D Systems, RRID: AB_2249965) or human ILT4/CD85d (1 µg/ml, AF2078, R&D systems, RRID: AB_355136) in the culture medium for 1 h at 37 °C with 5% CO_2_, fixed with 4% PFA in PBS for 1 h at RT, and heated in PBS containing 2 mM MgCl_2_ for 1 h at 67 °C. The treated cells were then incubated with biotin-SP-labeled donkey antibodies against goat IgG (0.7 µg/ml, 705-065-003, Jackson ImmunoResearch, RRID: AB_2340396) in TBS-T for 1 h at RT and with VECTASTAIN ABC-AP Staining Kit (AK-5000, Vector Laboratories, RRID: AB_2336792) diluted with TBS-T for 1 h at RT, followed by incubation with pNPP for 4 h at RT. The enzymatic reaction product of pNPP was measured using xMark Microplate Absorbance Spectrophotometer (Bio-Rad) and Microplate Manager 6 software (Bio-Rad) with absorbance at 405 nm wavelength.

### Primary culture

Hippocampal neurons obtained from WT or LOTUS-tg mice on embryonic day 17.5 (E17.5) were dissociated with 0.25% trypsin at 37 °C for 12 min and subsequently treated with 100 µg/ml DNase at 37 °C for 5 min. Dispersed cells were seeded in a 24-well dish (Greiner Bio-One) on poly-l-lysine (100 µg/ml, 163-19091, Wako)-coated glass coverslips (φ12 mm; Matsunami) or 6-well dishes at each concentration and incubated in neurobasal medium (21103, Gibco) containing 10% FBS and 10 mM 4-(2-hydroxyethyl)-1-piperazineethanesulfonic acid (Hepes)-NaOH (pH 7.3) for 3 h. Next, the medium was altered to a neurobasal medium containing 1×B-27 (17504-044, Gibco), 1×Glutamax (Gibco), 10 mM Hepes–NaOH (pH 7.3), and the cells were incubated at 37 °C with 5% CO_2_.

### PSD95 expression and cofilin dephosphorylation

Aβ peptide was added to cultured hippocampal neurons (2.5 × 10^5^ cells/well) at each concentration on DIV12 or DIV14 and incubated for 48 h (for PSD95) or 1 h (for cofilin dephosphorylation). For the experiments to examine the PirB antibody effect on the Aβ-induced PirB downstream pathway, the antibody against PirB (550348, BD Biosciences, RRID: AB_393627) or control rat IgG (6-001-A, R&D Systems, RRID: AB_10144734) was added at a final concentration of 3 µg/ml at the same time as Aβ peptide. Following each incubation, cells were rinsed with cold PBS and lysed using a lysis buffer containing 20 mM Tris–HCl (pH 7.6), 150 mM NaCl, 1 mM EDTA-NaOH (pH 8.0), 1% Nonidet P-40, 1 mM Na_3_VO_4_, 0.05 mM (*p*-amidinophenyl) methanesulfonyl fluoride (Wako, 019-26331), 0.1 U/mL aprotinin (Sigma-Aldrich, A6279) and the lysate was centrifuged at 20,000×*g* for 10 min at 4 °C. The supernatant was mixed with 4× Laemmli buffer (40% glycerol, 8% SDS, 250 mM Tris–Cl pH 6.8, 0.03% bromophenol blue) and heated at 100 °C for 7 min. The samples in Laemmli buffer were separated by SDS-PAGE and transferred onto a polyvinylidene fluoride membrane (Immobilon-P Membrane, IPVH00010, Millipore). The membrane was blocked with 5% skim milk in TBS-T for 1 h at RT, incubated sequentially with each primary antibody in TBS-T containing 5% skim milk for 1 h at RT with horseradish peroxidase (HRP)-labeled antibodies against mouse IgG, HRP-labeled antibodies against rabbit IgG in TBS-T containing 5% skim milk or 5% BSA for 1 h at RT, and immersed in chemiluminescent HRP substrate (ECL Western Blotting Detection Reagents, RPN2109, GE Healthcare Life Sciences) (Immobilon Western Chemiluminescent HRP Substrate, WBKLS0100, Millipore). The chemiluminescent signals were detected using ImageQuant LAS 4000 mini apparatus (GE Healthcare Life Sciences) or LAS-4000 multicolor apparatus (Fujifilm) and with ImageQuant TL software (GE Healthcare Life Sciences, RRID: SCR_014246).

### Hippocampal neuron transfection and spine imaging

Plasmid encoding EGFP was transfected into cultured hippocampal neurons (5.0 × 10^4^ cells/well) with Viafect (E4981, Promega) on DIV11for visualizing dendritic spine morphology in primary hippocampal neurons to assess for dendritic spine density. Approximately 24 h following transfection, Aβ was applied at each concentration and incubated for 48 h. The Aβ-treated cells were fixed at 4% PFA in PBS for 10 min at 37 °C. After washing with PBS, fluorescent images of the dendritic spine were acquired using a confocal microscope (TCS SP8; Leica) equipped with a 63× (NA, 1.4) oil-immersion objective and the LAS X software (Leica). Images were captured at a resolution of 1024 × 1024 pixels with a z-step of 0.3 µm. Cells with no abnormalities, including protrusion of cell membrane or fragmentation of dendrites, were evaluated.

### Protein purification

HEK293T cells (RRID: CVCL_0063), within 25 passages, were seeded (9 × 10^6^ cells/dish) on 145 mm cell culture dishes (639160, Greiner Bio-One), cultured in DMEM (08458-16, Nacalai Tesque) containing 10% FBS and 0.5% penicillin–streptomycin solution. Following 48 h culture, plasmid encoding Fc-SBP and LOTUS-Fc-SBP were transfected with lipofection reagent (Polyethylenimine Max, 24765, Polysciences) and cultured for an additional 4 days. The culture medium was ultracentrifuged at 117,000×*g* for 1 h. Subsequently, the supernatant was added to streptavidin beads (High Capacity Streptavidin Agarose Resin, 20361, Thermo Fisher Scientific). SBP-fused protein was eluted from the beads with PBS containing 2 mM biotin. These proteins were stored at − 80 °C until use. The protein sample was prepared with 4× Laemmli buffer (40% glycerol, 8% SDS, 250 mM Tris–Cl pH 6.8, and 0.06% bromophenol blue) containing 10% β-mercaptoethanol and boiled for 7 min to determine the concentration of purified proteins. Each sample was electrophoresed on a Tris–glycine SDS polyacrylamide gel, and the gel was incubated with Coomassie brilliant blue R-250 (031-17922, Wako Pure Chemical Industries). The intensity of the stained protein was measured for each concentration using an ImageQuant LAS 4000 mini instrument with ImageQuant TL software.

### Human LOTUS-LilrB2 ligand-receptor binding assay

Transfected Cos7 cells were treated with Fc-SBP or human LOTUS-Fc-SBP protein at each concentration and incubated for 1 h at 37 °C with 5% CO_2_. Following treatment, the cells were fixed with 4% PFA in PBS containing 2 mM MgCl_2_ for 1 h at RT, washed with PBS containing 2 mM MgCl_2_, and incubated for 1 h at 67 °C to inactivate endogenous AP. Subsequently, fixed cells were incubated sequentially with antibodies against SBP (0.04 μg/ml, sc-101595, Santa Cruz Biotechnology, RRID: AB_1128239) in 1% skim milk/TBS-T for 1 h at RT and with biotin-SP-labeled goat antibodies against mouse IgG (0.7 μg/ml, 115-065-003, Jackson ImmunoResearch, RRID: AB_2338557) in 1% skim milk/TBS-T for 1 h at RT, AP-conjugated avidin–biotin complex (AK-5000, Vector Laboratories, RRID: AB_2336792) was diluted to 1/5000 with TBS-T for 1 h at RT. The binding of the SBP-fused peptides was visualized with the enzymatic reaction product of NBT (11383213001, Roche) and BCIP, or detected quantitatively with that of pNPP. Digital images were captured using a BZ-8100 microscope (Keyence) equipped with a 10× objective lens. For the quantitative detection of protein binding, absorbance of the enzymatic reaction product of *p*-nitrophenyl phosphate (pNPP) (N2770-50SET, Sigma-Aldrich) was measured at 405 nm wavelength using the xMark Microplate Absorbance Spectrophotometer (Bio-Rad) and Microplate Manager 6 software (Bio-Rad). As previously reported, immunocytochemistry was used to confirm the cell surface expression of human LilrB2 under non-permeabilizing conditions (Kurihara et al. 2014).

## Results

### LOTUS inhibits the binding of Aβ to PirB

**Fig. 1:**
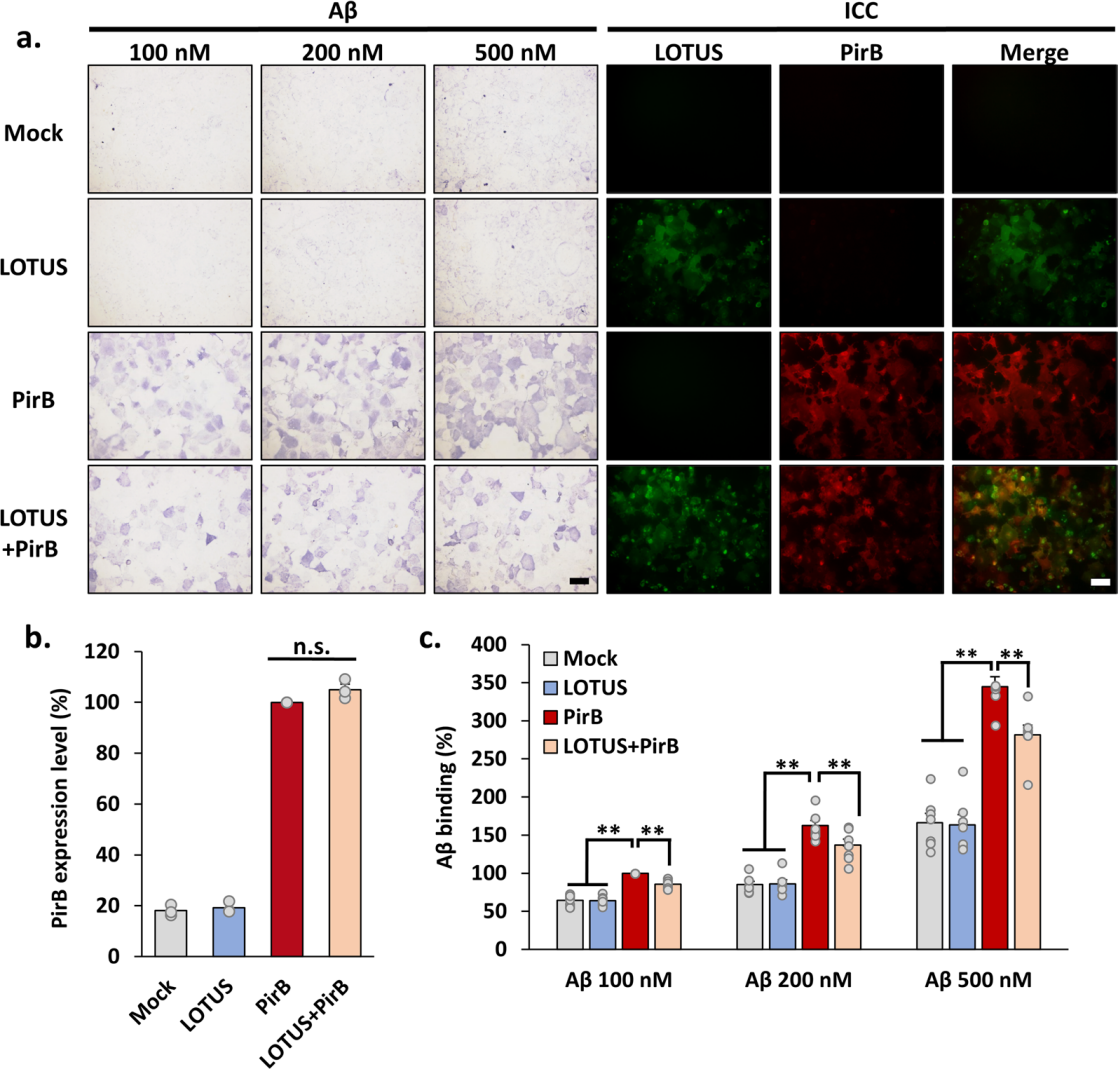
LOTUS inhibits Aβ-PirB binding. **a** Representative images of the binding assay in Cos7 cells overexpressing LOTUS or PirB. The cells were treated with biotin-conjugated-human Aβ and immunostained using LOTUS or PirB antibody without Aβ. Scale bars, 100 µm. **b** Quantification of cell surface expression level of PirB in Cos7 cells overexpressing PirB and/or LOTUS. Each expression level of PirB was normalized to the expression level in Cos7 cells overexpressing PirB alone, and the data are presented as mean ± standard error of the mean (SEM) from four independent cell culture preparations (n = 4, one way analysis of variance ANOVA post hoc Tukey test n.s., not significant). **c** Quantification of Aβ binding in Cos7 cells overexpressing LOTUS and/or PirB. Aβ binding levels in each group. Binding levels were normalized to the binding level of Aβ (100 nM) in Cos7 cells overexpressing PirB alone. The data are presented as mean ± SEM (n = 7, One-way ANOVA post hoc Tukey test *p < 0.05, **p < 0.01)

We performed a ligand-receptor binding assay using oligomerized biotin-LC-Aβ (Aβ) in Cos7 cells overexpressing LOTUS, PirB, or both to investigate whether LOTUS inhibits the function of PirB as an Aβ receptor. We immunocytochemically confirmed that LOTUS and PirB were expressed on the cell surface in Cos7 cells. PirB expression levels in cells expressing PirB alone or co-expressing LOTUS and PirB were nearly equivalent (Fig. 1a, b). Additionally, we confirmed that non-specific binding was extremely limited, and there was no obvious binding of Aβ to the cells overexpressing mouse LOTUS (Fig. 1a). While Aβ is specifically bound to the cells overexpressing PirB, as reported previously (Kim et al. 2013), the signaling from Aβ–PirB binding was reduced by approximately 30% in the cells co-expressing LOTUS and PirB at each concentration compared with PirB alone (Fig. 1a, c). These results suggest that LOTUS inhibits the binding of Aβ to PirB.

### LOTUS overexpression suppresses Aβ-induced cofilin dephosphorylation and downregulation of PSD95 expression

**Fig. 2:**
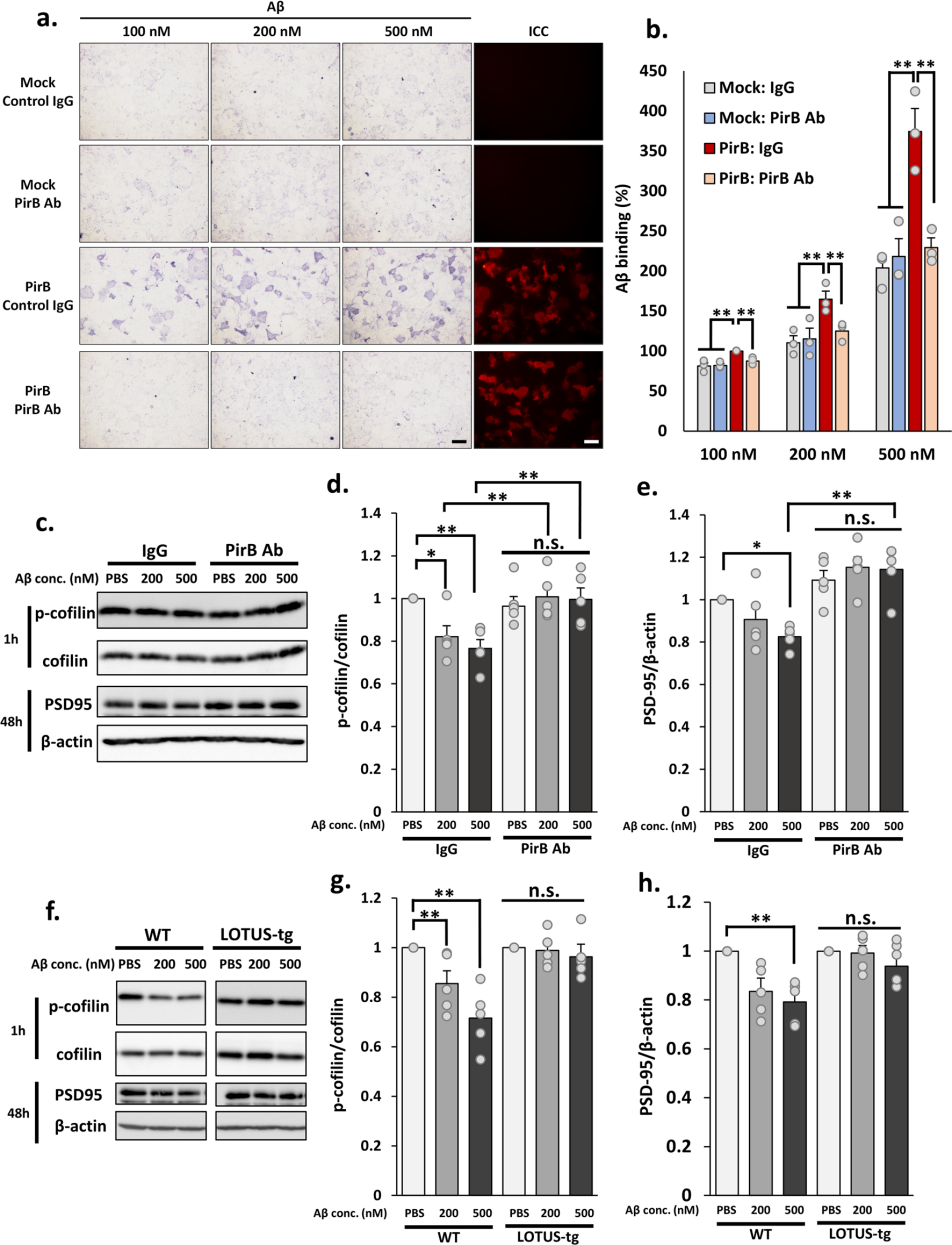
LOTUS overexpression suppresses Aβ-induced cofilin dephosphorylation and downregulation of PSD95 expression. **a** Analysis of functional blocking antibody against PirB. Representative images of the binding assay in Cos7 cells overexpressing PirB. The cells were treated with biotin-conjugated-human Aβ and PirB functional blocking antibodies and immunostained by PirB antibodies. Scale bars, 100 µm. **b** Quantification of Aβ binding with PirB overexpressed in Cos7 cells. Each binding level of Aβ was normalized to the binding level of Aβ (100 nM) in Cos7 cells overexpressing PirB treated with normal IgG, and the data are presented as mean ± SEM (n = 3, one-way ANOVA post hoc Tukey test *p < 0.05, **p < 0.01). **c** Representative images of western blotting of the phosphorylated coffin (p-cofilin), cofilin, PSD95, and β-actin in cultured hippocampal neurons of WT mice treated with Aβ and normal IgG or PirB functional blocking antibody. **d**, **e** Quantification of normalized p-cofilin and normalized PSD95 in **c**. Proportion of p-cofilin or PSD95 level was normalized to that treated with PBS and normal IgG-treated samples, and the data are presented as mean ± SEM (n = 5, Two-way ANOVA post hoc Tukey test *p < 0.05, **p < 0.01). **f** Representative images of immunoblots of the phosphorylated coffin (p-cofilin), cofilin, PSD95, and β-actin in cultured hippocampal neurons of WT or LOTUS-tg mice treated with Aβ. **g**, **h** Quantification of normalized p-cofilin and PSD95 in **c**. The level of p-cofilin or PSD95 was normalized to the level of control treated with PBS in samples of each genotype, and the data are presented as mean ± SEM (n = 5, One-way ANOVA post hoc Tukey–Kramer test *p < 0.05, **p < 0.01)

We first examined whether the antibody used in this study inhibited Aβ binding to PirB expressed in Cos7 cells (#550348, Kurihara et al. 2020). Treatment with the antibody completely inhibited Aβ binding to PirB expressed in Cos7 cells at each concentration in comparison with control IgG (Fig. 2a, b), suggesting that the PirB antibody completely blocks Aβ–PirB binding. PirB was found to mediate Aβ-dependent cofilin dephosphorylation and PSD95 downregulation (Kim et al. 2013; Qin et al. 2018). Cofilin is a primary actin-depolymerization factor and regulates spine density (Kommaddi et al. 2018). PSD95 is a scaffold protein expressed in the post-synaptic site, and its expression level in neurons is associated with synaptic plasticity (Migaud et al. 1998). To begin with, we investigated whether the PirB-neutralizing antibody inhibits Aβ-induced cofilin dephosphorylation in the primary cultured hippocampal neuron. While treatment using Aβ and control IgG decreased cofilin phosphorylation levels in an Aβ dose-dependent manner, treatment using Aβ and PirB antibodies did not alter cofilin phosphorylation levels (Fig. 2c, d). Furthermore, while cultured neurons treated with Aβ and control IgG for 48 h exhibited PSD95 downregulation in an Aβ dose-dependent manner, treatment with Aβ and PirB antibodies suppressed PSD95 downregulation (Fig. 2c, e). These findings demonstrate that PirB mediates this Aβ-induced cofilin dephosphorylation and PSD95 downregulation in primary cultured hippocampal neurons.

Next, we assessed whether LOTUS overexpression inhibits Aβ-induced cofilin dephosphorylation and PSD95 downregulation in primary cultured hippocampal neurons obtained from LOTUS-tg mice. While treatment with Aβ in neurons from WT mice decreased cofilin phosphorylation and PSD95 downregulation in an Aβ dose-dependent manner, Aβ treatment did not affect these aspects in neurons obtained from LOTUS-tg mice (Fig. 2f–h). These findings suggest that LOTUS suppressed Aβ-induced cofilin dephosphorylation and PSD95 downregulation in primary cultured hippocampal neurons in a manner similar to that of PirB-neutralizing antibody.

### LOTUS overexpression suppresses Aβ-induced dendritic spine elimination

**Fig. 3:**
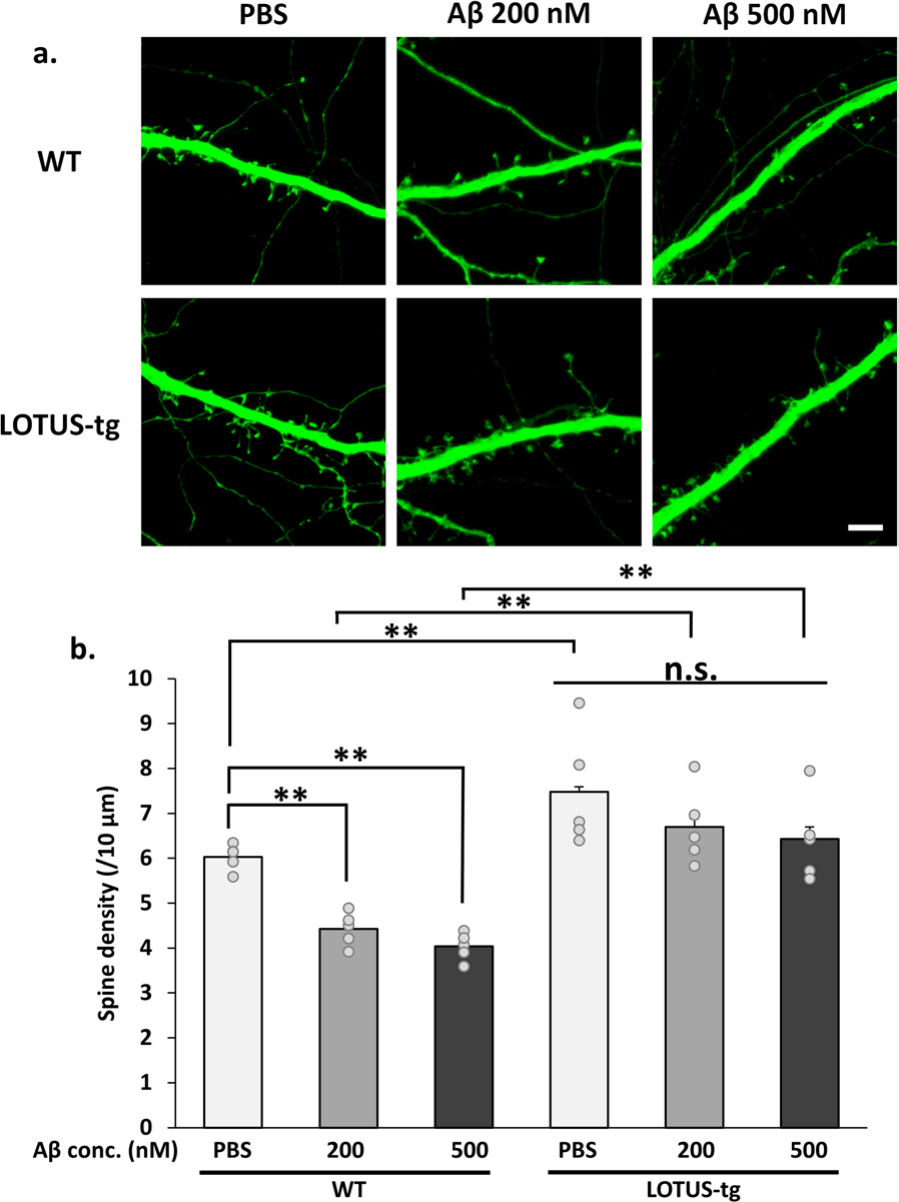
LOTUS overexpression suppresses Aβ-induced decrease of spine density. **a** Representative images of dendritic spines of cultured hippocampal neurons from WT or LOTUS-tg mice after 48 h of treatment with Aβ. Scale bars, 5 μm. **b** Quantification of spine density of each dendrite per 10 µm. The data are presented as mean ± SEM from five independent cell culture preparations (n = 5, 5 or more cells were analyzed at each experiment, Two-way ANOVA post hoc Tukey test *p < 0.05, **p < 0.01)

Synaptic density decreases in the brains of patients with AD and AD model mice (Selkoe 2002; Querfurth and LaFerla 2010). Because LOTUS suppressed Aβ-induced cofilin dephosphorylation and PSD95 downregulation, as depicted in Fig. 2, it is likely concluded that LOTUS contributes to inhibiting the Aβ-induced decrease in spine density. We investigated and compared the spine density in primary cultured hippocampal neurons from WT and LOTUS-tg mice to test this theory following Aβ treatment. Although the spine density in LOTUS-tg mice without Aβ treatment was approximately 20% higher than that in wild-type mice, we found that Aβ treatment reduced spine density in neurons obtained from WT mice (Fig. 3). In contrast, Aβ treatment did not reduce spine density in neurons obtained from LOTUS-tg mice (Fig. 3). These results demonstrate that LOTUS suppresses the Aβ-induced decrease in spine density.

### Human LOTUS binds to LilrB2 and inhibits Aβ binding to LilrB2

**Fig. 4:**
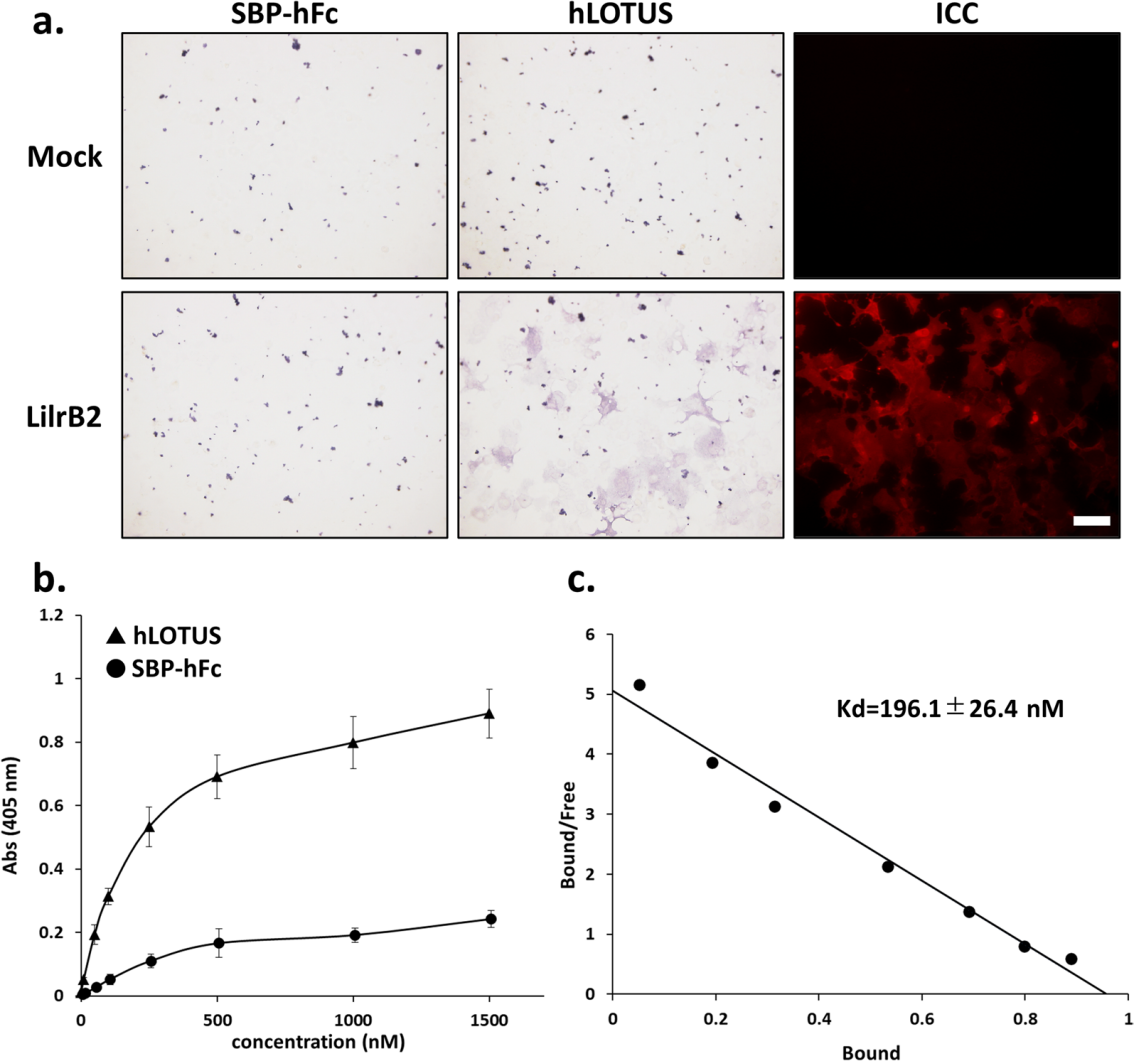
Binding ability human soluble LOTUS and LilrB2. **a** Representative images of the binding assay using soluble human LOTUS protein (LOTUS-Fc-SBP, 500 nM) in LilrB2-overexpressing Cos7 cells and immunocytochemistry with the antibody against LilrB2. Scale bars, 100 μm. **b**, **c** Saturation curves (**b**) and scattered analysis (**c**) of binding control protein or soluble human LOTUS to LilrB2 overexpressed in Cos7 cells. The data are presented as mean ± SEM from three independent cell culture preparations (n = 3). The Kd value was estimated to be 196.1 ± 26.4 nM

Our study showed that mouse LOTUS binds to mouse PirB and inhibits Nogo66-PirB binding (Kurihara et al. 2020). However, whether human LOTUS (hLOTUS) inhibits LilrB2 function is yet to be determined. Therefore, to test whether hLOTUS binds to LilrB2, we first conducted a ligand-receptor binding assay using hLOTUS with human Fc and SBP tagged at C-terminus (hLOTUS-Fc-SBP) in LilrB2-overexpressing Cos7 cells (Fig. 4a). The cell surface expression of LilrB2 was determined by immunocytochemistry using antibodies against LilrB2 (Fig. 4a). LOTUS-Fc-SBP was found to be specifically bound to LilrB2 overexpressed in Cos7 cells (Fig. 4a, b). Scatchard analysis showed that the dissociation constant (Kd) value for the binding of hLOTUS-Fc-SBP to LilrB2 was approximately 196.1 ± 16.4 nM (Fig. 4c).

**Fig. 5:**
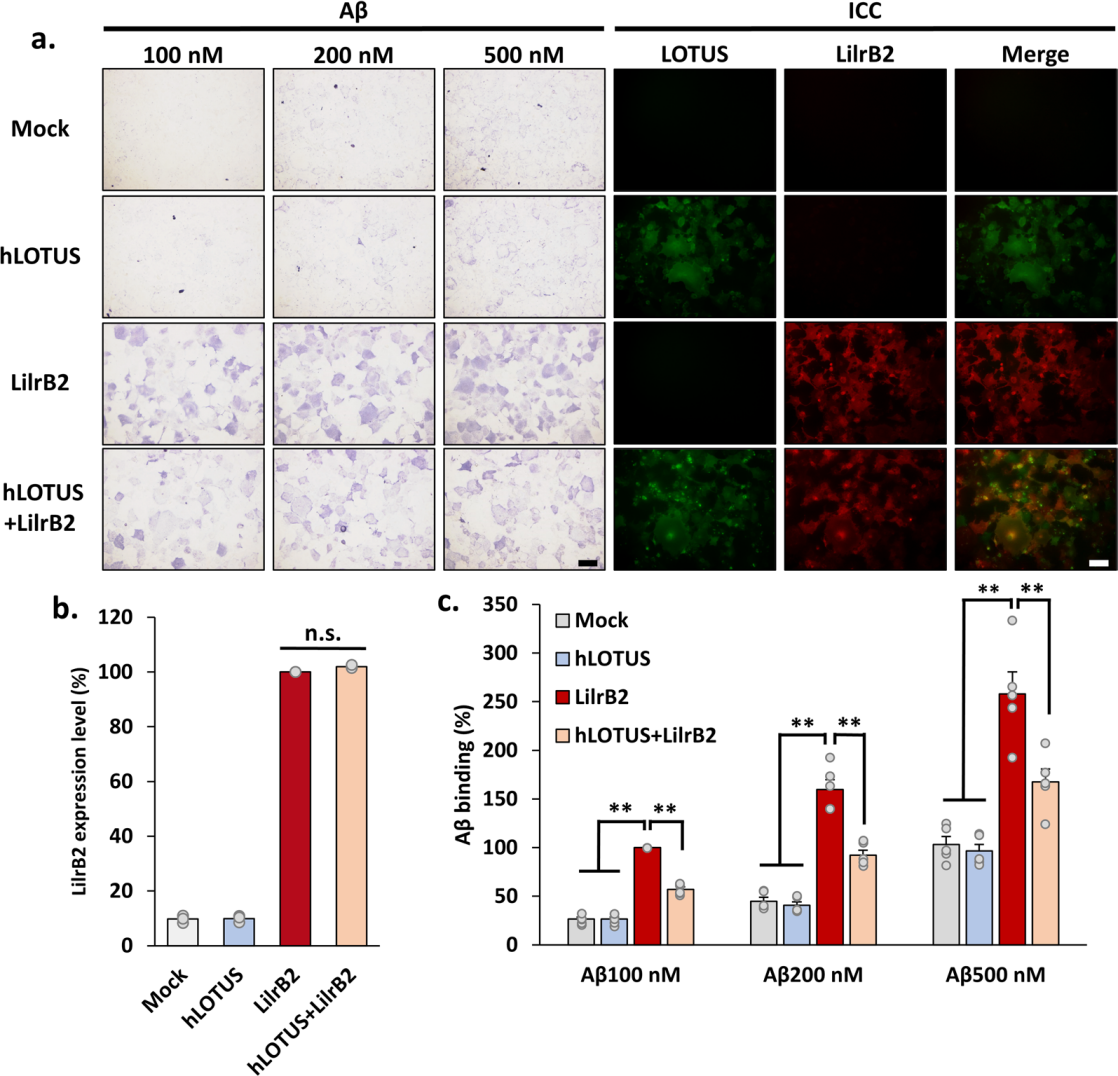
Human LOTUS inhibits Aβ binding to LilrB2. **a** Representative images of the binding assay in Cos7 cells overexpressing human LOTUS or LilrB2. The cells were treated with biotin-conjugated-human Aβ and immunostained using LOTUS or PirB antibody without Aβ. Scale bars, 100 µm. **b** Quantification of cell surface expression level of LilrB2 in Cos7 cells overexpressing human LOTUS and/or LilrB2. Each expression level of LilrB2 was normalized to the expression level in Cos7 cells overexpressing LilrB2 alone, and the data are presented as mean ± SEM from four independent cell culture preparations (n = 4, one-way ANOVA post hoc Tukey test n.s., not significant). **c** Quantification of Aβ binding in Cos7 cells overexpressing LOTUS or LilrB2. Aβ binding levels in each group were normalized to the binding level of Aβ (100 nM) in Cos7 cells overexpressing PirB alone, and the data are presented as mean ± SEM (n = 5, One-way ANOVA post hoc Tukey test *p < 0.05, **p < 0.01)

Next, to evaluate whether hLOTUS inhibits Aβ-LilrB2 binding, we conducted a ligand-receptor-binding assay using oligomerized biotin-LC-tagged Aβ peptide (Aβ) to LilrB2 and hLOTUS overexpressed in Cos7 cells (Fig. 5a). The cell surface expression of hLOTUS and/or LilrB2 was determined by immunocytochemistry using antibodies against hLOTUS and/or LilrB2 applied to the unfixed Cos7 cells. There was no difference between the LilrB2 expression level of cells expressing LilrB2 alone or co-expressing LOTUS and LilrB2 (Fig. 5b). As previously reported, we showed that Aβ was clearly bound to overexpressed LilrB2 (Fig. 5a, c) (Kim et al. 2013). Aβ binding was reduced by approximately 40% in the cells co-expressing hLOTUS and LilrB2 at each concentration in comparison with cells expressing LilrB2 only (Fig. 5a, c). These results imply that human LOTUS also binds to LilrB2 with a nanomolar scale and has an inhibitory role against Aβ-LilrB2 binding.

## Discussion

Aβ contributed to the loss of synapse and dendritic spine in the early phases of AD (Mucke et al. 2000; Selkoe 2002). PirB has been implicated in this pathology as a receptor of Aβ and has thus been regarded as a novel therapeutic target against AD (Kim et al. 2013). In this study, we demonstrated that LOTUS was a promising inhibitory agent against Aβ-induced synapse elimination via Aβ-PirB binding, cofilin dephosphorylation, and PSD95 downregulation in cultured hippocampal neurons.

PirB is a type I transmembrane glycoprotein, which is composed of six extracellular Ig-like domains (D1–D6), its transmembrane region, and four immunoreceptors tyrosine-based inhibitory motifs (ITIMs) or ITIM-like sequences in its intracellular region (Takai 2005). Nogo-66 binds to two amino-terminal Ig-like domains (D1/D2) and the four carboxy-terminal Ig-like domains (D3–D6) in PirB, and the binding affinity of Nogo-66 for D3–D6 is higher than that for D1/D2 (Matsushita et al. 2011). However, Aβ binds to D1/D2 domains (Kim et al. 2013; Qin et al. 2018). Our previous study showed that LOTUS completely blocks the binding of Nogo-66 to PirB (Kurihara et al. 2020). We found that LOTUS partially inhibited the binding of Aβ to PirB (Fig. 1a, c). Thus, we hypothesized that LOTUS interacts with D3–D6 domains of PirB with a stronger binding affinity than that for D1/D2 domains and that this interaction may result in an allosteric inhibition of Aβ binding to PirB. However, further studies are required to identify the binding domain of LOTUS to PirB to understand the detailed molecular mechanism in inhibiting Aβ binding to PirB by LOTUS.

As reported previously, PirB mediates Aβ-induced cofilin dephosphorylation and PSD95 downregulation (Kim et al. 2013; Qin et al. 2018). We demonstrated that LOTUS overexpression suppressed Aβ-induced cofilin dephosphorylation and PSD95 downregulation in cultured hippocampal neurons (Fig. 2f–h). These suppressive effects by LOTUS overexpression were shown in a similar manner by the PirB-neutralizing antibody (Fig. 2c–e). This shows that Aβ-induced cofilin dephosphorylation and PSD95 downregulation through PirB are inhibited by LOTUS overexpression, and LOTUS may have an inhibitory role against Aβ–PirB signaling. Moreover, LOTUS overexpression suppressed the Aβ-induced decrease in spine density (Fig. 3). Thus, LOTUS is believed to suppress Aβ-induced synapse elimination via the blockade of cofilin dephosphorylation and PSD95 downregulation. Therefore, LOTUS may suppress Aβ-induced synaptotoxicity, although LOTUS shows partial inhibition in Aβ binding to PirB (Fig. 1).

Nogo–NgR1 signaling inhibits synapse formation via the RhoA–Rho–associated protein kinase signal, and NgR1 knockdown increases spine formation (Willis et al. 2012; Karlsson et al. 2016). Furthermore, NgR1 reportedly mediates an Aβ–induced decrease in synaptic density and dysfunction (Zhao et al. 2017a, b). Therefore, NgR1 has also been considered a critical receptor associated with synaptic failure in AD. We demonstrated that LOTUS completely inhibits Nogo–NgR1 signaling (Sato et al. 2011; Kurihara et al. 2014, 2017; Kawakami et al. 2018a, b). Our previous studies showed that LOTUS is involved in spine formation, as mice lacking the *lotus* gene showed reduced spine density (Nishida et al. 2021). Furthermore, we found that LOTUS-tg mice had increased hippocampal spine density compared to wild-type mice (Nishida et al. in submission, unpublished data). In this study, spine density was significantly increased in primary cultured neurons generated from LOTUS-tg mice compared to those generated from wild-type mice, suggesting that a biological function of LOTUS is to promote spine formation. On the other hand, it has been reported that PirB suppresses synapse formation, and conversely, suppression of PirB promotes synapse formation (Djurisic et al. 2013; Bochner et al. 2014; Vidal et al. 2016; Albarran et al. 2021), indicating that suppression of PirB by LOTUS may promote synapse formation.

Thus, PirB- and NgR1-mediated synapse elimination may be suppressed by LOTUS, which could exert a synergistic inhibitory effect on Aβ-induced AD pathology. Further studies are required to determine if LOTUS inhibits Aβ-induced synapse elimination via NgR1.

Aβ induces neuronal cell death in AD pathology (Haass and Selkoe 2007). PirB is involved in neuronal cell death (Zhao et al. 2017a, b; Wang et al. 2018; Qin et al. 2018; Zhang et al. 2021), and the increase in PirB worsens neuronal apoptosis following oxygen and glucose deprivation (OGD) injury (Zhao et al. 2017a, b). Blockade of PirB ligand binding by PirB ectodomain improves neuronal apoptosis following OGD injury in vitro and cerebral cortex ischemic stroke in vivo (Wang et al. 2018). Recent studies have demonstrated that the PirB blockade with antagonistic peptides improved Aβ-induced neuronal cell death (Qin et al. 2018; Zhang et al. 2021). These results suggest that counteracting PirB may be crucial to protect against neuronal cell death. Therefore, the suppression of PirB function by LOTUS may also have a neuroprotective effect against Aβ–induced neuronal cell death.

Many studies using AD model mice and autopsied brains of patients with AD have demonstrated that synaptic dysfunction is relevant to cognitive impairment (Selkoe 2002; Querfurth and LaFerla 2010; Kim et al. 2013; Zhao et al. 2017a, b; Long and Holtzman 2019). Because our previous study showed that LOTUS plays a physiological role in synapse formation and learning and memory functions (Nishida et al. 2021), considering the physiological function of LOTUS in synapse formation, antagonistic action of LOTUS against Aβ-induced synapse elimination may have a possible function to improve the pathology and cognitive impairment in patients with AD. Therefore, more research is required to evaluate how LOTUS affects cognitive function in AD model animals and alters LOTUS expression in patients with AD.

Finally, this study explicitly demonstrated the molecular interaction between human LOTUS and LilrB2, the human homolog of PirB. LilrB2 has been reported to act as an Aβ receptor (Kim et al. 2013; Smith et al. 2019). Human LOTUS bound to LilrB2 at a nanomolar scale (Fig. 4), and human LOTUS inhibited the binding of Aβ to LilrB2 (Fig. 5). These results suggest that LOTUS is valuable for the pharmacological treatment of AD, even in humans. However, which domains of human LOTUS and LilrB2 interact with each other remains unclear. Therefore, additional investigation is required to understand the detailed molecular mechanism in which human LOTUS inhibits the binding of Aβ to LilrB2.

## Conclusion

In conclusion, we demonstrated that LOTUS inhibits the binding of Aβ to PirB in rodents or LilrB2 in humans and that LOTUS suppressively regulates Aβ-induced intracellular responses, including dephosphorylation of cofilin and the reduction of PSD95, and that LOTUS suppresses the Aβ-induced synaptotoxic phenomenon, such a decrease in spine density. Therefore, this study provides insight into a novel therapeutic strategy for the blockade of Aβ pathological function.

## Data Availability

All data generated or analyzed during this study are included in this published article.

## Abbreviations

ANOVA: Analysis of variance
AP: Alkaline phosphatase
BSA: Bovine serum albumin
CNS: Central nervous system
DMEM: Dulbecco’s modified eagle’s medium
FBS: Fetal bovine serum
HRP: Horseradish peroxidase
Ig: Immunoglobulin
ITIM: Immunoreceptor tyrosine-based inhibitory motif
LOTUS: Lateral olfactory tract usher substance
MAI: Myelin-associated inhibitor
NgR1: Nogo receptor-1
PCR: Polymerase chain reaction
PFA: Paraformaldehyde
Pir: Paired immunoglobulin-like receptor
pNPP: *p*-Nitrophenyl phosphate
RRID: Research resource identifier
SBP: Streptavidin binding protein
SEM: Standard error of the mean
TBS: Tris-buffered saline
